# A strategy of tumor treatment in mice with doxorubicin-cyclophosphamide combination based on dendritic cell activation by human double-stranded DNA preparation

**DOI:** 10.1186/1479-0556-8-7

**Published:** 2010-11-01

**Authors:** Ekaterina A Alyamkina, Valeriy P Nikolin, Nelly A Popova, Evgenia V Dolgova, Anastasia S Proskurina, Konstantin E Orishchenko, Yaroslav R Efremov, Elena R Chernykh, Alexandr A Ostanin, Sergey V Sidorov, Dmitriy M Ponomarenko, Stanislav N Zagrebelniy, Sergey S Bogachev, Mikhail A Shurdov

**Affiliations:** 1 Novosibirsk State University, Novosibirsk, Russia; 2 Institute of Cytology and Genetics, Siberian Branch, Russian Academy of Sciences, Novosibirsk, Russia; 3 Institute of Clinical Immunology, Siberian Branch, Russian Academy of Medical Sciences, Novosibirsk, Russia; 4 Municipal Hospital, Oncology Department, Novosibirsk, Russia; 5 Regional Oncologic Dispensary, Irkutsk, Russia; 6 LLC Panagen, Gorno-Altaisk, Russia

## Abstract

**Background:**

Immunization of mice with tumor homogenate after combined treatment with cyclophosphamide (CP) and double-stranded DNA (dsDNA) preparation is effective at inhibition of growth of tumor challenged after the treatment. It was assumed that this inhibition might be due to activation of the antigen-presenting cells. The purpose was to develop improved antitumor strategy using mice. We studied the combined action of cytostatics doxorubicin (Dox) plus CP with subsequent dsDNA preparation on tumor growth.

**Methods:**

Three-month old CBA/Lac mice were used in the experiments. Mice were injected with CP and human dsDNA preparation. The percentage of mature dendritic cells (DCs) was estimated by staining of mononuclear cells isolated from spleen and bone marrow 3, 6, and 9 days later with monoclonal antibodies CD34, CD80, and CD86. In the next set of experiments, mice were given intramuscularly injections of 1-3 × 10^5 ^tumor cells. Four days later, they were injected intravenously with 6-6.7 mg/kg Dox and intraperitoneally with 100-200 mg/kg CP; 200 mkg human DNA was injected intraperitoneally after CP administration. Differences in tumor size between groups were analyzed for statistical significance by Student's t-test. The MTT-test was done to determine the cytotoxic index of mouse leucocytes from treated groups.

**Results:**

The conducted experiments showed that combined treatment with CP and dsDNA preparation produce an increase in the total amount of mature DCs *in vivo*. Treatment of tumor bearers with preparation of fragmented dsDNA on the background of pretreatment with Dox plus CP demonstrated a strong suppression of tumor growth in two models. RLS, a weakly immunogenic, resistant to alkalyting cytostatics tumor, grew 3.4-fold slower when compared with the control (p < 0.001). In experiment with Krebs-2 tumor, only 2 of the 10 mice in the Dox+CP+DNA group had a palpable tumor on day 16. The cytotoxic index of leucocytes was 86.5% in the Dox+CP+DNA group, but it was 0% in the Dox+CP group.

**Conclusions:**

Thus, the set of experiments we performed showed that exogenous dsDNA, when administered on the background of pretreatment with Dox plus CP, has an antitumor effect possibly due to DC activation.

## Background

The most effective antitumor treatment is currently achieved by chemotherapeutic agents that abrogate tumor cells [1]. Despite this, chemotherapy is virtually without influence on life expectancy of patients with certain cancers. With this in mind, novel strategies for treating malignancies are being developed in experiments and applied in clinical setting. These are targeted towards potentiation of immune mechanisms of antitumor defense [2,3]. The conventional vaccines are utilized, also those based on the pathogen-associated molecular patterns (PAMPs) of bacteria, including endo/exotoxins of bacterial origin, and CpG DNA preparations [4-12].

Dendritic cells (DCs), which are capable of activating T-lymphocytes, including naive T-cells, have an important role in triggering and development of the adaptive immunity [9,13,14]. Mature DCs that express MHC antigens of class I and class II, also the various costimulatory molecules CD40, CD54, CD80, and CD86 are capable of only presenting foreign antigens within the MHC complex [15-21].

Search of novel inducers of antitumor immunity has been intense over the past years. It has been revealed that mammalian double-stranded DNA (dsDNA) induces both humoral and adaptive immune responses [15,22,23]. This induction is provided by the action of dsDNA preparations primarily on professional antigen-presenting cells. This process enfolds via the TLR-independent pathway and is mainly due to activation of TANK-binding kinase-1, TBK1 [22-27]. As a result of internalization of exogenous DNA, DCs up-regulate expression and secretion of type I interferon-beta (INF-β) [22,25]. In addition, dsDNA induces complete DC maturation, by stimulating expression of cofactor molecules on cell wall needed for development of the adaptive immunity [15].

Cyclophosphamide (CP) is a drug widely applied in the clinic to treat cancers. The effect is predominantly based on direct cytotoxic action on tumor cells resulting in their lysis. CP has an influence on CD4+CD25+FoxP3 regulatory T cells. Regulatory T cells accumulate predominantly in the tumor microenvironment and lymphoid organs [28] where they suppress activation and proliferation of the other immune cells [28-32]. When administered at moderate doses, CP not only induces a reduction in numbers of regulatory T cells [33-35], also diminishes their functionality [32,34], thereby allowing to reduce the intensity of the immunosuppressive background in tumor microenvironment and to activate the antitumor immune response [31,32,35]. The effect of CP on various DC subsets was manifest as enhancement of antitumor immunity [36-38].

It has been amply demonstrated that under the combined effect of CP and dsDNA preparation (CpG DNA, for example), the immune system is stimulated and tumor growth is suppressed [**for reference, see 9**]. The therapeutic effect is synergic in that cytostatic preferentially decreases the amount of regulatory T cells in the tumor microenvironment and/or directly kills tumor cells, while dsDNA preparation stimulate maturation and activity of cells of the adaptive immunity [9,39].

There are chemotherapeutic agents capable of potentiating immunogenicity of tumor cells directly at the level of the organism. Doxorubicin (Dox), idarubucin, and mitoxanthrone, cytostatics of the antracycline series, are of this kind. A relevant observation was that induction of exposure of the protein calreticulin on cell surface of dying cells is required for activation of the antitumor immune system [40,41]. Calreticulin is a calcium-binding lectin chaperone, mainly represented on endoplasmic membrane. Its exposure on cell surface of dying tumor cells acts as an "eat me" signal for removal by neighboring phagocytic cells [40,42] and facilitates thereby their almost instantaneous capture [41]. The combination of Dox with cytostatic drugs (CP plus paclitaxel) and whole-cell vaccines was highly effective in enhancing antitumor response in transgenic mice [43].

Here, we demonstrate that human exogenous dsDNA preparation induces maturation of mouse spleen and bone marrow DCs *in vivo*. To evaluate the efficacy of vaccination with human dsDNA preparation, we chose a strategy whereby mice were treated with preparation of fragmented dsDNA on the background of pretreatment with Dox plus CP. This strategy provided the presence of tumor antigens thanks to the *in vivo *abrogation of tumor by the combined action of cytostatics. The subsequently injected dsDNA preparation induced effective DC maturation. This strategy demonstrated a considerable delay in tumor growth. Cytotoxic test provided evidence indicating that in the blood there appeared a cell population with high, up to 86.5%, cytotoxic activity against cells of the challenged tumor.

## Methods

### Laboratory animals and tumor models

Three-month old CBA/Lac mice (henceforth designated as CBA) that were bred at the animal facility of the Institute of Cytology and Genetics (IC&G), the Siberian Branch of the Russian Academy of Sciences, were used in experiments. Mice in groups of 10 were housed in plastic cages in a well-illuminated room. They had free access to food and water. All experiments were performed in accordance with protocols approved by the Animal Care and Use Committee of the IC&G.

Krebs 2 ascitic carcinoma is a strain-nonspecific tumor derived from epithelial cells; all inbred mouse strains can be challenged with Krebs 2 tumor cells. When challenged subcutaneously (s.c.) or intramuscularly (i.m.), it grows as solid nodes. It is weakly immunogenic for mice of all strains. It does not give rise to metastases [44].

Lymphosarcoma LS is strain-specific to CBA mice; it was induced in them by nitrosomethylurea, passages in ascitic form. When challenged i.m., it grows as solid nodes. It develops in 100% of challenged mice, does not regress spontaneously. It is subjected to apoptosis under the effect of alkylating antitumor agents. It metastasizes to liver, kidneys, lungs. Lymphosarcoma RLS-40 is a version of LS tumor. It is resistant to alkylating compounds [45,46].

Mice were injected i.m. into the right hind limb with tumor cells at a dose of 1-3 × 10^5 ^cells/mouse. The tumors were allowed to grow to solid nodes. As soon as tumor became palpable, about 7 days after challenge, it size was measured with calipers every 1-2 days. Tumor size was calculated by multiplying the three perpendicular diameters. Differences in tumor size between groups were analyzed for statistical significance by Student's t-test.

### DNA preparation

Human DNA preparation was isolated from the placentas of healthy women using a phenol-free method. It was fragmented in an ultrasonic disintegrator at a frequency of 22 kHz to obtain a mixture of DNA fragments with a size 200-6,000 bp. The human DNA was a pharmacopeian preparation "Panagen" (Registration certificate Medical Drugs of Russia No. 004429/08 of 09.06.2008). This preparation does not contain steroid hormones and RNA. It gives negative PCR results for hepatitis B virus DNA, hepatitis C virus RNA, HIV DNA, HIV RNA. The DNA preparation does not contain histones and polysaccharides; it is also endotoxin-free.

### Estimation of DC maturity *in vivo*

Mice were injected with CP (Veropharm, Russia) at 200 mg/kg and 200 mkg of human dsDNA preparation 1 day (on the day of CP injection), 3, 4, and 5 days after CP treatment. Three, 6, and 9 days later, the fraction of mononuclear cells (MNCs) was isolated from spleen and bone marrow. MNCs were isolated also from untreated mice. Every group consisted of 4-6 mice. The experiment was repeated twice.

Mice were anesthetized and sacrificed by cervical dislocation. Femurs and tibias were removed and bone marrow cells were flushed from them by RPMI-1640 (Sigma-Aldrich) medium. Washed bone marrow cells (DC precursors) were suspended in RPMI-1640. Spleen contents were scraped out with pincers into Petri dishes and resuspended in PBS. The obtained cell suspension was applied onto 3 ml ficoll 400 (Farmaceg) - urografin (Schering) gradient, centrifuged (5810R, Eppendorf) at 1,500 rpm for 30 min. MNCs were collected, washed and precipitated. Cell residue was suspended in RPMI-1640, the number of cells was counted and diluted to 2 × 10^5 ^in 200 μl of medium.

The percentage of mature spleen and bone marrow DCs was estimated by staining with monoclonal antibodies CD34-PerCP, CD80-FITC, and CD86-PE (Santa Cruz). Cells were analyzed on a flow cytofluorometer BD FACSAria (BD Biosciences). Additional file 1 is a dot plot figure of the event gating for CP+DNA group.

Statistics was based on estimates of the number of mature DCs relative to the total number of isolated MNCs.

### Schedule for treatment with exogenous dsDNA preparation after administration of cytostatics Dox plus CP

CBA mice were given an i.m. challenge with 10^5 ^RLS-40 tumor cells. Four days later, they were injected intravenously (i.v.) with 6.7 mg/kg Dox (Veropharm, Russia) and i.p. with 100 mg/kg CP; 200 mkg human DNA was injected i.p. after 30 min, then 2 and 3 days after CP administration. Mice were assigned to three groups (n = 10) according to treatment schedule: 1) challenged tumor + PBS injections (control); 2) Dox + CP; 3) Dox + CP + DNA.

CBA mice were given 3 × 10^5 ^Krebs-2 tumor cells injected i.m.. Four days later, they were administered i.v. 6 mg/kg Dox and i.p. 200 mg/kg CP; 200 mkg human DNA was administered i.p. 30 min after CP, also 2, 3, and 5 days after it. Assignment of mice to groups, with 10 in each, was as follows: 1) challenged tumor + PBS injections (control); 2) Dox + CP; 3) DNA; 4) Dox + CP + DNA. The experiment was done in triplicate.

The dosages of Dox and CP were the conventionally used for chemotherapy in the clinic, 100-200 mg/kg for CP and 6-7 mg/kg for Dox. The DNA preparation was used at 200 mkg/mouse/injection. This amount has been defined in experiments [39].

### MTT test

Mice of all the 4 groups and one untreated mouse were sacrificed by decapitation on day 16 after tumor Krebs-2 challenge. Blood (200-500 μl) was drawn into tubes containing 800 μl PBS with 50 mM EDTA. Blood cells were precipitated by centrifugation (5810R, Eppendorf) at 1,500 rpm for 5 min at room temperature; erythrocytes from cell residue were lysed with 0.15 M ammonium chloride.

In *in vitro *cytotoxicity study, Krebs-2 cells were plated in 96-well plates (3 × 10^4 ^cells/well), and mouse leucocytes were added at a 1:1 ratio. Cells were incubated in RPMI-1640 medium supplemented with gentamycin sulfate (100 mkg/ml) and maintained at 37°C for 18 h in 5% CO_2 _atmosphere. After incubation, MTT (Sigma) was added to a final concentration of 0.5 mg/ml and cells were cultured for additional 3 h. Cells were centrifuged (5810R, Eppendorf) at 4,000 rpm for 10 min. Medium was collected, precipitated blue formasan crystals were dissolved in 100 μl DMSO. Optical density was determined on a Multiscan RC at 570 nm, background was subtracted at 620 nm. Measurements were done for three samples. The MTT-test was repeated twice for different experiments.

The standard formula was applied to calculate the percentage of dead cells:

$$\left(\%\right)=\left[\text{1}–\left({\text{D}}_{\text{e}+\text{t}}–{\text{D}}_{\text{e}}\right)/{\text{D}}_{\text{t}}\right]\times \text{1}00,$$

D_e+t_, the optical density value in wells with cells from mice of the treated groups incubated with tumor cells;

D_e_, the optical density value in wells with effectors (leucocytes);

D_t_, the optical density value in wells with targets (tumor cells).

The cytotoxic index (CI) was expressed as the difference between the percentage of dead cells in the treated groups and the untreated mouse.

## Results

Our previous study has demonstrated that a preparation of human fragmented dsDNA stimulated maturation of mouse DCs in culture [47]. The salient finding was that the dsDNA preparation was just as effective at induction of DC maturation as the standard inducer TNF-α. The obtained mature DCs loaded with antigen during maturation were used in the comparative test. A marked antitumor effect was observed after vaccination with DCs irrespective of the type of maturation inducer [47].

**Figure 1:**
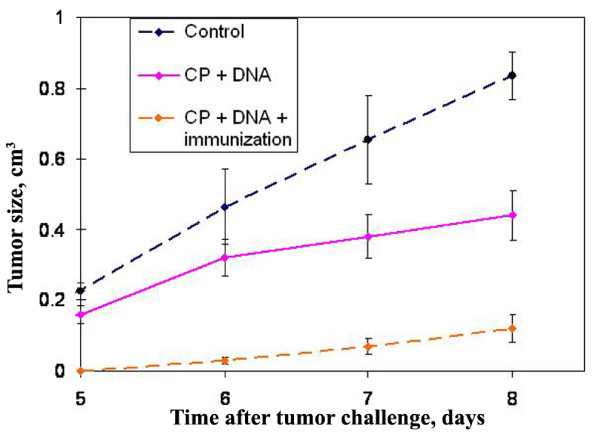
**Time course of Krebs-2 tumor growth in mice (mean ± SEM)**. Time course of Krebs-2 tumor growth in mice (mean ± SEM). Mice received 200 mg/kg CP and human DNA at a total dose 4.5-6 mg. After this treatment, one group of mice was pre-immunized with Krebs-2 tumor antigens by a s.c. injection of 20 × 10^6 ^repeatedly thawed-frozen tumor cells. The control group was injected with saline. Every group consisted of 10 mice. 10^6 ^Krebs-2 tumor cells were challenged i.m. after the treatment. Immunization enhanced the suppressive effect on tumor growth [31].

Previous experimental sets with Krebs-2 tumor demonstrated that immunization of mice with tumor homogenate after combined treatment with CP and dsDNA preparation is effective at inhibition of growth of tumor challenged after the treatment (Figure 1) [39]. Proceeding on reported observations [14,39,47], we assumed that this inhibition may be due to the inducer effect of dsDNA on DC maturation *in vivo *that causes effective presentation of antigens of tumor lysate and activates antitumor mechanisms of the adaptive immunity.

The results provided evidence indicating that the described antitumor activity was not related to natural killer cells [39]. This appeared plausible, because, to our knowledge, NK-cells neither displayed nor enhanced antigen-specific cytotoxicity associated with tumor homogenate immunization [48,49].

### Effect of dsDNA preparation on maturation of spleen and bone marrow DCs in vivo

**Figure 2:**
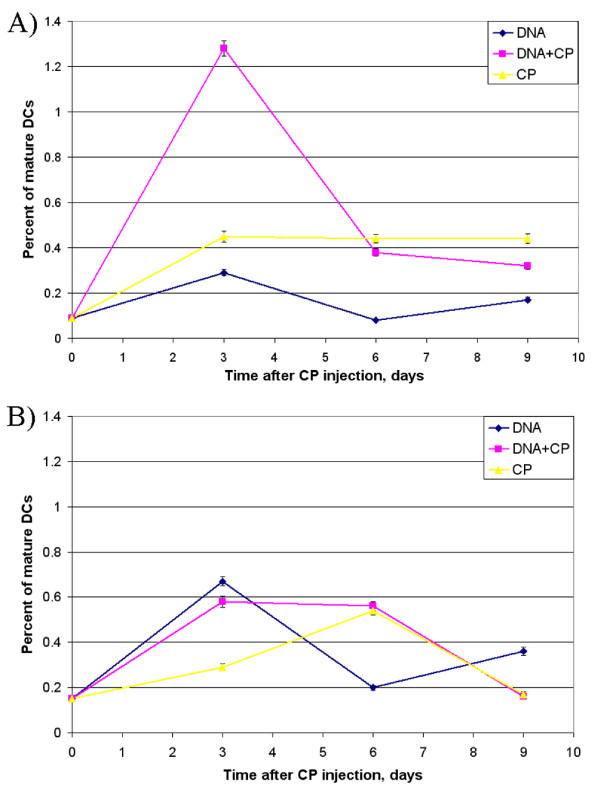
**Time course of maturation of mouse DCs from spleen (A) and bone marrow (B) after treatment with CP and dsDNA preparation (mean ± SEM)**. Time course of maturation of mouse DCs from spleen (A) and bone marrow (B) after treatment with CP and dsDNA preparation (mean ± SEM). 0 represents the number of mature DCs in untreated mice. Mice were injected with CP 200 mg/kg and 200 mkg of human dsDNA preparation 1 day (on the day of CP injection), 3, 4, and 5 days after CP treatment. Three, 6, and 9 days later, the fraction of MNCs was isolated from spleen and bone marrow. Every group consisted of 4-6 mice. The experiment was repeated twice.

To obtain assurance that dsDNA has an inducer effect on DCs *in vivo*, a set of experiments was undertaken. Mice were treated with CP 200 mg/kg followed by 200 mkg human dsDNA preparation administration 1, 3, 4, and 5 days after CP injection. The number of mature CD34-CD80+CD86+ DCs among spleen and bone marrow cells was estimated 3, 6, and 9 days after CP had been injected (Figure 2).

The peak of spleen DC maturation was 3 days after combined DNA+CP treatment. This peak was followed by a decrease in the number of mature DCs presumably due to migration of cells to lymph nodes and other sites of their specific localization. Mouse groups treated with an agent alone, CP or dsDNA preparation, showed no marked increase in the number of mature DCs.

The peak of bone marrow DC maturation in the DNA and DNA+CP groups was also on day 3. In the case of DNA+CP treatment, the interval during which DCs retained mature phenotype and were able to effectively present antigen was longer, several days. DNA alone caused a transient rise in level of mature DCs. In the CP group, the number of mature DCs in bone marrow reached the maximum by day 6 only, thereafter it decreased to the initial level.

Thus, the conducted experiments showed that combined treatment with CP and dsDNA preparation produces an increase in the total amount of mature DCs. This was associated with an increase in the time during which mature DCs persisted at high levels.

### Effect of inhibition of tumor growth induced by Dox+CP+DNA treatment

Our previous studies have demonstrated that the CP+DNA combination was statistically superior to each treatment modality alone [39,50]. From comparisons of schedules, the standard with additional immunization with tumor homogenate, it followed that the presence of specific antigens further enhanced the suppression effect on tumor growth. There were reasons for suggesting that the integration of cytostatics with dsDNA preparation may be a treatment modality for enhancing regression of established tumors.

According to the data in the literature a combination of cytostatics is superior to each modality alone [51,52]. Two-three potent drugs are usually combined in the clinic. In the current study, we did not strive to control the effectiveness of a drug as monotherapeutic agent. We were rather interested in the antitumor action of DNA preparation when used in combination with cytostatics Dox and CP.

Proceeding on the combined cytotoxic action of Dox and CP, also on the course of changes in DC maturation *in vivo*, a set of experiments was designed. The idea was to superimpose the effects of released tumor antigens and of their capture by DCs. Mice bearing established tumors were treated on day 4 with Dox and CP, thereafter they were injected with human dsDNA preparation. As known [41,53], Dox provides the exposure of the cell surface endoplasmic protein calreticulin that acts as an "eat me" signal and mediates the phagocytosis of tumor cells by DCs. CP abrogates tumor cells, thereby increasing the amount of free tumor antigens that, thanks to the "eat me" signal, are uptaken promptly, and presented by DCs. The induction of DC maturation is the necessary condition for antigen presentation on the surface of DCs. In the following experiments, we chose dsDNA preparation as a maturation stimulus.

**Figure 3:**
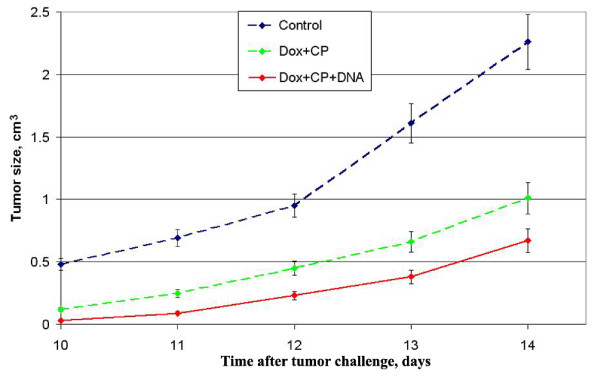
**Time course of RLS tumor growth in mice that received combined treatment with Dox, CP, and dsDNA preparation (mean ± SE)**. Time course of RLS tumor growth in mice that received combined treatment with Dox, CP, and dsDNA preparation (mean ± SE). Mice were given i.m. injections of 10^5 ^RLS-40 tumor cells. Four days later, they were injected i.v. with 6.7 mg/kg Dox and i.p. with 100 mg/kg CP; 200 mkg human DNA was injected i.p. after 30 min, then 2 and 3 days after CP administration. The control group was injected with PBS. Every group consisted of 10 mice.

Using this schedule, a strong suppression of tumor growth was observed in two murine models. The size of RLS, a weakly immunogenic, resistant to alkalyting cytostatics tumor, on day 14 was 3.4-fold smaller (p < 0.001) in the Dox+CP+DNA group compared with the control (Figure 3). The difference in RLS size on day 14 between the groups Dox+CP and Dox+CP+DNA was 1.5-fold (p < 0.1).

**Figure 4:**
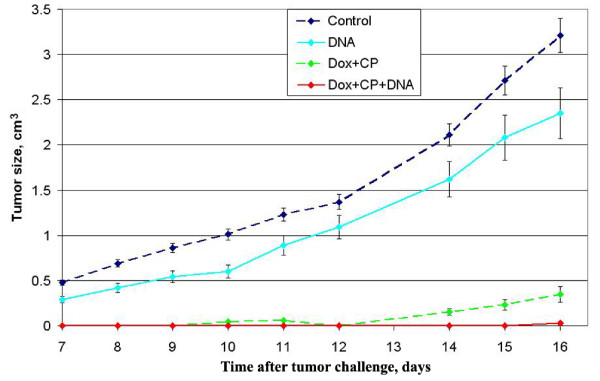
**Time course of Krebs-2 tumor growth in mice treated with Dox, CP, and dsDNA preparation (mean ± SE)**. Time course of Krebs-2 tumor growth in mice treated with Dox, CP, and dsDNA preparation (mean ± SE). Mice were given 3 × 10^5^Krebs-2 tumor cells injected i.m.. Four days later, they were administered i.v. 6 mg/kg Dox and i.p. 200 mg/kg CP; 200 mkg human DNA was administered i.p. 30 min after CP, also 2, 3, and 5 days after it. The control group was injected with PBS. Every group consisted of 10 mice. The experiment was done in triplicate.

Krebs-2 tumor growth was effectively suppressed as compared to the control in both Dox+CP and Dox+CP+DNA groups (p < 0.001) (Figure 4). A tumor burden was of measurable size 16 days after treatment in 9 of the 10 mice in the Dox+CP group, but only in 2 of the 10 mice tumor was palpable on day 16 in Dox+CP+DNA group. There was a 14-fold significant difference (p < 0.005) in tumor size on day 14 between the Dox+CP and Dox+CP+DNA groups. Injection of dsDNA preparation alone slightly suppressed Krebs-2 tumor growth, the difference from the control being significant, however (p < 0.05).

The schedule for DNA preparation administration differed slightly from the one we applied to estimate the efficacy of DC maturation *in vivo*. DNA was injected at the time when the number of mature DCs was maximum.

We used CP at high doses since evaluation of therapeutic combined action of CP and dsDNA did not demonstrate enhancement of antitumor effect with low-dose CP (data not shown).

### Estimation of cytotoxic activity of blood cells in mice with Krebs-2 tumor after combined treatment with Dox+CP+dsDNA preparation

The experimental results provided evidence for activation of the antitumor immune response *in vivo*. Supporting data of the MTT test were required. For this purpose, treated mice bearers of Krebs-2 tumor were sacrificed 16 days after treatment. All the mouse groups could be monitored at the same time, on day 16 for the presence of cytotoxic cells. This became feasible because tumors reached the size that led to lethal development in the control group. Tumor size in the treated groups attained a statistically significant difference from the control by this time.

Peripheral blood was monitored for the appearance of cells showing antitumor cytotoxic activity. Krebs-2 cells derived from ascitic version of tumor served as targets (Table 1).

**Table 1:** Cytotoxic activity of leucocytes in MTT test.

	Absorption	Dead cells, %	CI
Tumor cells (targets)	1.363		

Leucocytes (effectors)	0.34		

Untreated mouse	1.42	20.9	

Control (tumor only)	1.706	-0.2	**-21.1**

Dox+CP	1.424	20.5	**-0.4**

DNA	0.815	65.1	**44.2**

Dox+CP+DNA	0.239	107.4	**86.5**

The cytotoxic index (CI) was expressed as the percentage of dead cells relative to their number in an untreated mouse. It was 86.5% in the Dox+CP+DNA group, consistent with the time course of tumor growth (Figure 4). It was 0% in the Dox+CP group, although there was a considerable suppression of tumor growth. This may be attributed to the direct cytostatic effect on tumor growth of the kind that does not enhance cytotoxic activity of circulating leucocytes. Such was the case, because there was no DNA stimulus for DC maturation and ultimate development of antigen-specific immune responses. dsDNA preparation itself raised cell cytotoxic activity to 44.2%, but tumor growth was suppressed just slightly.

## Discussion

Tumors have unique properties allowing them to elude immune defense. To begin with, they are genetically flexible owing to the incessantly activated repair-recombination system of tumor cells [32]. Second, tumor tissue takes advantage of the properties of regulatory T lymphocytes. Third, a tumor is, as a rule, weakly immunogenic and this makes the more difficult for the immune system to reveal malignized cells and to eradicate them. Modulation or elimination of these three properties of tumors would create conditions favorable for the immune system to eliminate neotransformed cells [1,13,54,55].

The current increasing trend is to affect tumor tissue by using in a defined sequence two modalities, a chemotherapeutic (a cytostatic, most commonly CP) followed by an immunotherapeutic [21,56-58]. This strategy is fully consistent with the idea how tumor tissue may be affected. To recapitulate, CP directly attacks tumor cells, it also causes a decrease in the numbers of regulatory T cells and reduces their functionality [21,32,34,35,58,59], thereby improves the efficacy of immune-based therapies directed at stimulation/enhancement of antitumor immune responses.

Recent studies on the chemotherapeutic effects of antracyclines have established that Dox, for example, transposes calreticulin to the cell surface. This protein may play the role of surveillance "eat me" signal and mediate the phagocytosis of tumor cells by DCs. As a result, tumor immunogenicity is enhanced [41,53].

Cytostatics (CP and Dox) in combination with immunotherapeutics (DNA activators) allow to develop improved antitumor strategy. CP directly injures tumor cells, concomitantly switches regulatory T cells off. Dox also abrogates tumor and renders tumor cell debris immunogenic. The DNA activated immune system kills the remaining neotransformed cells at the time when the regulatory T-lymphocytes are inactive and tumor is defenseless.

In the current experiments, we relied on the ability of dsDNA to induce complete DC maturation *ex vivo *reasonably expecting that this would augment their stimulatory activity in an allogenic mixed lymphocyte culture [14,22,47]. It was a reasonable assumption that dsDNA would manifest its stimulatory action on DCs at the level of the whole organism. The suggestion that antitumor dsDNA activity [39,50,60] is due to precisely endogenous DC activation and development of the adaptive immune response lent credibility to our line of reasoning.

We determined the extent to which spleen and bone marrow derived DCs were mature and followed the time course of changes in their quantitative accumulation after different treatments. Given the results, a schedule for combined Dox plus CP, which form apoptotic/necrotic debris, plus dsDNA preparation was developed. Strongest suppression of tumor growth was achieved with this schedule and an optimal sequence of administration of each modality. Its effectiveness was confirmed by the MTT test estimates. The suppression effect on tumor growth was, indeed, due to both damaging action of cytostatics and formation of a pool of cytotoxic cells. Importantly, challenged tumors virtually stopped growing when chemotherapeutic agents were combined with dsDNA preparation.

## Conclusions

Thus, the set of experiments we performed showed that exogenous dsDNA, when administered on the background of pretreatment with Dox plus CP, has an antitumor effect possibly due to DC activation. The effect may be also explained by DC-mediated activation of cytotoxic T-lymphocytes [37,38]. Crucial here are the mature phenotype of DCs, i.e. their antigen-presenting ability, and the real presence of tumor antigens achieved by combined treatment with Dox and CP.

The described approach to therapy of cancers appears promising. Injections of dsDNA preparation may be well integrated into classical schedules of chemotherapy.

## Competing interests

The authors declare that they have no competing interests.

## Authors' contributions

EAA carried out the mice experiments and performed the statistical analysis. VPN carried out the mice experiments, performed the analysis, and interpreted the data. NAP participated in the design of the study and performed the statistical analysis. EVD carried out the mice experiments and performed the statistical analysis. ASP carried out the mice experiments and drafted the manuscript. KEO participated in the design of the study. YRE performed the analysis. ERC performed the analysis and interpreted the data. AAO participated in the design of the study and helped with drafting the manuscript. SVS helped in the data interpretation. DMP participated in the study design. SNZ participated in the study design and helped with the data interpretation. SSB conceived the study, participated in its design, and coordinated and drafted the manuscript. MAS participated in the study design and coordination. All authors read and approved the final manuscript.

## Supplementary Material

Additional file 1 **Dot plot figure**. Dot plot figure of the event gating for CP+DNA group. Click here for file
