# Supplementary figures and images for: A strategy of tumor treatment in mice with doxorubicin-cyclophosphamide combination based on dendritic cell activation by human double-stranded DNA preparation
Source: Genet Vaccines Ther. 2010 Nov 1;8:7. doi: 10.1186/1479-0556-8-7 (PMC2987767; doi:10.1186/1479-0556-8-7)

Cell  
number

CD34

CD80

CD86

Isotype control

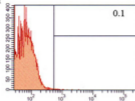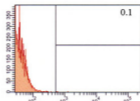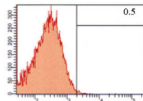

0 day

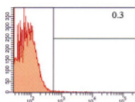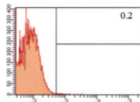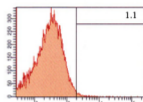

3 day

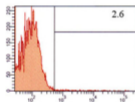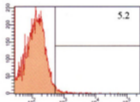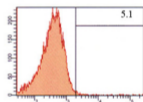

6 day

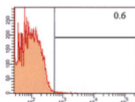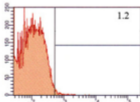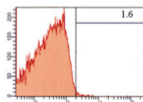

9 day

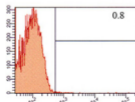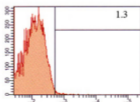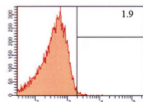

Fluorescence intensity

Supplement: Additional file 1 — Dot plot figure. Dot plot figure of the event gating for CP+DNA group. [file 1479-0556-8-7-S1.PDF]
